# Cloning and Characterization of a Hybridoma Secreting a 4-(Methylnitrosamino)-1-(3-pyridyl)-1-butanone (NNK)-Specific Monoclonal Antibody and Recombinant F(ab)

**DOI:** 10.3390/toxins5030568

**Published:** 2013-03-19

**Authors:** Heather Wanczyk, Tolga Barker, Debra Rood, Daniel I. Zapata, Amy R. Howell, Stewart K. Richardson, John Zinckgraf, Gregory P. Marusov, Michael A. Lynes, Lawrence K. Silbart

**Affiliations:** 1 Department of Animal Science, University of Connecticut, Storrs, CT 06268, USA; E-Mails: heather.wanczyk@uconn.edu (H.W.); debra.rood@uconn.edu (D.R.); 2 National Institutes of Allergy and Infectious Diseases, National Institutes of Health, Bethesda, MD 20892, USA; E-Mail: barkertt@niaid.nih.gov; 3 New York Medical College, New York, NY 10162, USA; E-Mail: dzapata84@gmail.com; 4 Department of Chemistry; University of Connecticut, Storrs, CT 06268, USA; E-Mails: amy.howell@uconn.edu (A.R.H.); stewart.richardson@uconn.edu (S.K.R.); 5 Immucell, Portland, ME 04101, USA; E-Mail: jzinckgra@yahoo.com; 6 Department of Molecular and Cell Biology, University of Connecticut, Storrs, CT 06268, USA; E-Mails: gregmarusov@gmail.com (G.P.M.); michael.lynes@uconn.edu (M.L.); 7 Department of Allied Health Sciences, CANR, 358 Mansfield Road, University of Connecticut, Storrs, CT 06269-2101, USA

**Keywords:** TSNAs, F(ab), NNK, monoclonal antibody, 2A cleavage

## Abstract

Smokeless tobacco products have been associated with increased risks of oro-pharyngeal cancers, due in part to the presence of tobacco-specific nitrosamines (TSNAs) such as 4-(methylnitrosamino)-1-(3-pyridyl)-1-butanone (NNK). These potent carcinogens are formed during tobacco curing and as a result of direct nitrosation reactions that occur in the oral cavity. In the current work we describe the isolation and characterization of a hybridoma secreting a high-affinity, NNK-specific monoclonal antibody. A structurally-related benzoyl derivative was synthesized to facilitate coupling to NNK-carrier proteins, which were characterized for the presence of the *N*-nitroso group using the Griess reaction, and used to immunize BALB/c mice. Splenocytes from mice bearing NNK-specific antibodies were used to create hybridomas. Out of four, one was selected for subcloning and characterization. Approximately 99% of the monoclonal antibodies from this clone were competitively displaced from plate-bound NNKB conjugates in the presence of free NNK. The affinity of the monoclonal antibody to the NNKB conjugates was *K*_d_ = 2.93 nM as determined by surface plasmon resonance. Free nicotine was a poor competitor for the NNKB binding site. The heavy and light chain antibody F(ab) fragments were cloned, sequenced and inserted in tandem into an expression vector, with an FMDV Furin 2A cleavage site between them. Expression in HEK 293 cells revealed a functional F(ab) with similar binding features to that of the parent hybridoma. This study lays the groundwork for synthesizing transgenic tobacco that expresses carcinogen-sequestration properties, thereby rendering it less harmful to consumers.

## 1. Introduction

Smokeless tobacco products have been listed as IARC Group 1 human carcinogens since 1987 based primarily upon human epidemiological data [1,2]. Although there are many known or suspected carcinogens present in smokeless tobacco, the precise nature of its carcinogenicity remains undefined. Among the most carcinogenic chemicals found in these products are polycyclic aromatic hydrocarbons such as benzo(a)pyrene, metals such as cadmium and lead, radioactive elements such as polonium-210, and a group of tobacco-specific nitrosamines (TSNAs) that include 4-(methylnitrosamino)-1-(3-pyridyl)-1-butanone (NNK), *N*'-nitrosonornicotine (NNN), and 4-(methylnitrosamino)-1-(3-pyridyl)-1-butanol (N-NAL). *N*-NAL is a metabolite of NNK and can be detected in the urine and plasma of smokers [3]. NNN is structurally related to NNK and is likewise formed from the nitrosation of nicotine. Numerous animal studies have confirmed the carcinogenic properties of several members of the TSNA family, with NNN and NNK among the most potent and prevalent constituents in the class [4]. The mechanism of carcinogenicity for these compounds involves P-450 mediated α-hydroxylation reactions that bioactivate the parent molecule to an electrophile capable of reacting with DNA and other macromolecules. Extra-hepatic P-450 enzymes such as CYP2A13 and CYP2A6 have been shown to be involved in α-hydroxylation reactions of NNK resulting in the methylation or pyridyloxobutylation of deoxyguanosine residues, forming the *O*^6^-alkylguanosine and *O*^6^-[4-oxo-4-(3-pyridyl)butyl]guanine (O6-pobG) DNA adducts, respectively [3,5]. It is widely accepted that covalent modification of DNA leads to mutations being fixed in the genome following either aberrant repair processes or disruption of DNA replication in the S-phase of the cell cycle, thereby predisposing the affected cells to tumorigenesis. 

Alternatively, epigenetic mechanisms involving nicotinic receptor ligation have been proposed for some nicotine derivatives which act as tumor promoters. NNK can stimulate cell division upon ligation of acetylcholine nicotinic receptors [6] including the α7 nicotinic acetylcholine receptor [7] which drives proliferation of certain human cell lines of epithelial origin. The role of these receptors in human oro-pharyngeal epithelial cell cancers has been elucidated in several different studies, providing evidence that NNK ligation leads to activation of transcription factors necessary for malignant cellular transformation. [8,9,10]. Preventing the interaction of such chemicals with the relevant receptors and/or activating enzymes through immune-mediated sequestration may reduce the risk of epithelial cancers of the oral cavity, pharynx and esophagus.

The public health community resoundingly supports smoking cessation, even if FDA approved nicotine-only patches, lozenges, gums or water are needed to facilitate the behavior change. However, many smokers who are trying to quit prefer the convenience and social acceptability of smokeless tobacco products, regardless of what public health officials recommend. Although substituting one tobacco habit for another is highly controversial, smokeless tobacco products possess only about 2% of the mortality risk associated with cigarette smoking, and paradoxically, about half the risk of oral cancer [11,12]. Switching to smokeless tobacco is also among the most effective methods of smoking cessation, and was recently measured at 73% effective [13]. Based on evidence accumulating over the past decade, these risks can be reduced even further as the result of smokeless tobacco companies working to lower TSNA levels in their products. Taken together, this “harm-reduction” approach, although distasteful to many, may represent an important component in our overall efforts to mitigate the devastating effects of smoking. The current work represents an extension of this general approach. If the residual carcinogenicity of smokeless tobacco products can be further reduced, these products may one-day be equivalent to other FDA-approved nicotine delivery systems. 

Passive administration of poly- or monoclonal antibodies specific for a variety of small molecules has been used clinically to ameliorate or eliminate toxicity in humans afflicted with an intentional or accidental drug overdose, or simply due to overmedication [14]. Antibody-mediated interception has shown therapeutic efficacy against nicotine, cocaine, methamphetamine and phencyclidine, among others [15]. Furthermore, this approach can reduce the absorption of carcinogens [16,17], and protect sensitive target tissues from chemically-mediated toxicity caused by colchicine, oleander [18,19,20], digitalis and related compounds, and endogenous digitalis-like substances present in pre-eclampsia [21], and provide therapeutic benefits for patients addicted to drugs of abuse [22,23]. In this context, either active immunization or passive administration of an immunotherapeutic agent is administered depending on whether or not persistent immunity is needed (e.g., for nicotine) *vs.* therapeutic intervention following accidental or intentional overdose (e.g., cocaine, PCP, digitalis *etc*.). One concern regarding active immunity to nicotine is that in instances where an individual is intractably addicted to nicotine, anti-nicotine antibodies will likely result in heavier product consumption to override the capacity of the antibody binding sites, thereby exposing the individual to higher concentrations of toxic/carcinogenic chemicals [15]. For this reason, NNK has been targeted for immune-mediated sequestration due to the fact that it is one of the most abundant carcinogens in smokeless tobacco and its sequestration and elimination will not interfere with nicotine delivery.

Producing smokeless tobacco products that sequester their own carcinogens is technically feasible using recombinant antibody approaches. As a first step, high affinity recombinant binding sites must be created. One way this can be accomplished is through the use of viral 2A self-cleaving peptide sequences that are 19–33 AAs in length and contain a functional conserved motif (D (V/I) EXNPGP) [24]. These peptides, derived from the viral family picornaviridae, mediate autonomous intra-ribosomal self-processing of multiple proteins from a single open reading frame (ORF) [25,26]. A commonly used 2A peptide originating from Foot and Mouth Disease Virus (FMDV), facilitates this process through “ribosomal skipping” or “stop carry-on translation”, whereby the peptide bond between the glycine and proline located at the *C*-terminal of the 2A sequence is cleaved due to interaction of the amino acids with the ribosomal exit tunnel [27,28,29]. After liberation of the 2A sequence from the downstream protein, translation by the eukaryotic ribosome resumes. All that remains on this protein is the proline from the 2A sequence, which has been shown to have no effect on activity or expression [30]. A furin cleavage site is commonly inserted at the *N*-terminus of the 2A motif in order to remove the remainder of the peptide from the upstream protein. In the current work, the 2A peptide derived from FMDV was used to create an anti-NNK recombinant binding. This approach enabled the parent hybridoma to be expressed as a single chain antibody fragment. This antibody format is beneficial because its smaller structure facilitates ease of cloning into expression vectors, elicits minimal immunogenicity and is rapidly cleared from the circulation [31]. Following production, the recombinant antibody, delivered by the appropriate vector or admixed with the product, may be used to passively protect the oral cavity prior to or during smokeless tobacco use. NNK bioactivation will be prevented once the high-affinity antibody binds to its target and the complex is harmlessly eliminated through expectoration. Future studies are needed to determine the safety and efficacy of this approach in animal models and humans. 

A long-term goal of the current proposal is to produce transgenic tobacco that allows the free passage of nicotine from the product, but blocks the bioavailability of carcinogens like NNK by immuno-interception. The expression of recombinant antibodies in tobacco has already proven to be technically feasible and will allow for the large-scale production of therapeutic antibodies at relatively low costs, making this approach particularly attractive to individuals seeking cost-effective tobacco harm reduction products. 

## 2. Results

**Figure 1:**
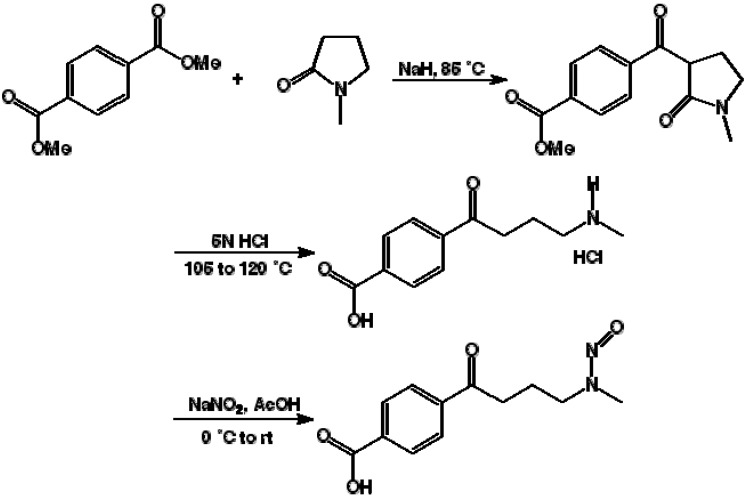
Synthetic scheme for synthesis of 4-(methylnitrosamino)-1-(3-pyridyl)-1-butanone NNKB as modified from [32]. Synthesis of the NNK mimic was performed through a series of several steps and resulted in a structure similar to NNK. The only differences were the substitution of the pyridyl group of NNK for a benzyl group, to which a carboxyl group was subsequently attached.

**Figure 2:**
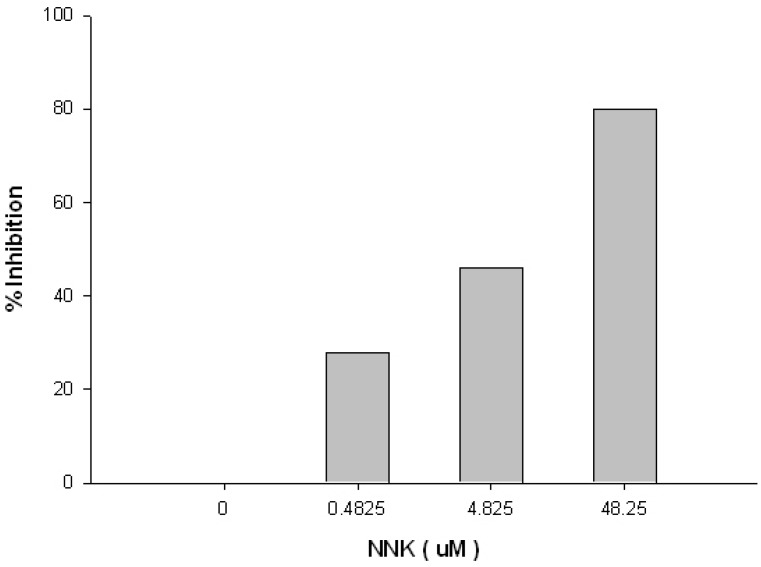
Competitive inhibition of mouse antiserum from NNKB-BSA immunized mice when pre-incubated with free NNK. 96-well plates were coated with NMBA:Ova. Free NNK was used as a competitor for antibody binding. The antibodies were derived from mice inoculated with the NNKB-BSA conjugate. Mouse anti-serum was competitively displaced from binding NMBA: Ova coated plates by up to 80% when incubated with 48.2 μM of free NNK.

The synthetic scheme described in Figure 1 yielded the NNKB derivative, which could be conveniently coupled to carrier proteins using standard carbodiimide procedures. The resulting NNKB-carrier protein conjugates were characterized by UV absorption spectra, *N*-nitroso content by the Greiss reaction, and protein concentration, yielding conjugates with 7–12 NNKB substitutions per mole (data not shown). The NNKB-BSA conjugates were chosen for subsequent immunization of mice, with a booster immunization given four weeks later. The anti-sera were then tested by ELISA for anti-NNKB binding activity. All four mice immunized with NNKB-BSA conjugates demonstrated varying degrees of anti-NNKB binding activity (data not shown). Subsequent competition experiments in which antisera was pre-incubated with free NNK before addition to the ELISA plates indicated that approximately 80% of the binding to NNKB-carrier protein coated wells could be inhibited when antisera was pre-incubated with 48.25 μM of free NNK (Figure 2).

**Figure 3:**
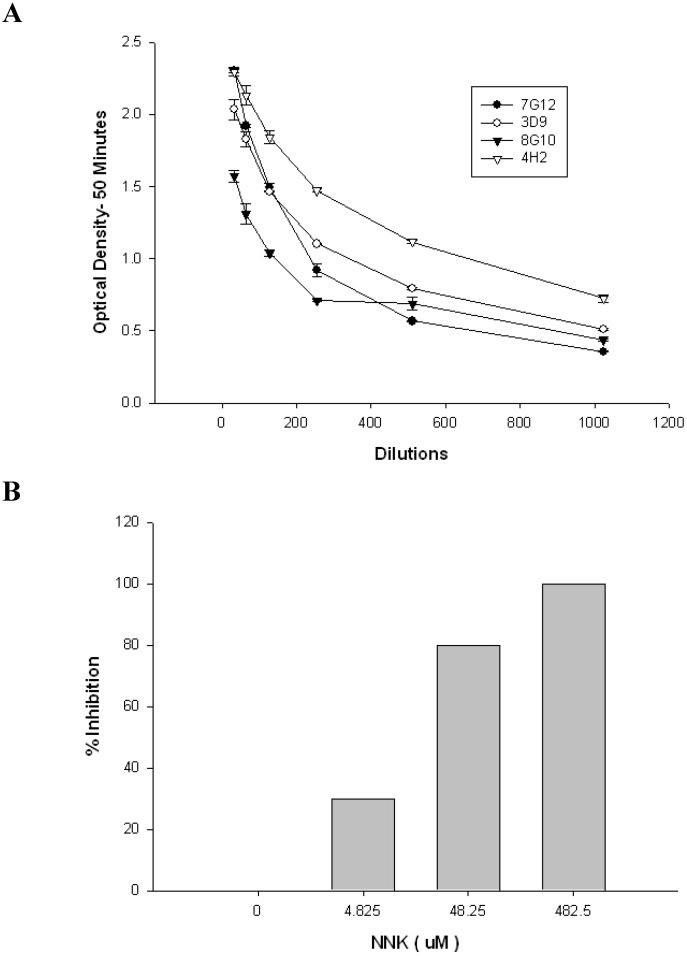
(**A**) Titration curves for four NNKB binders. Supernatants from four candidate hybridomas (4H2, 8G10, 7G12, 3D9) were tested for their ability to bind to plates coated with NNKB:Ova. Doubling dilutions ranging from 1:16–1:1024 were performed and ELISA screening confirmed that 4H2 bound to NNKB conjugates with the highest affinity at a time point of 50 min; (**B**) Competitive Inhibition of mAb 4H2 when pre-incubated with NNK. A 10 mg/mL (48.25 mM) stock solution of NNK was diluted to final concentrations of 4.825 μM, 48.25 μM and 482.5 μM and each was co-incubated with a 1: 160 dilution of 4H2 (1:1 ratio) at room temperature for 30 min. Solutions were then applied to 96-well plates coated with NNKB. The greatest inhibition of 4H2 was seen at a concentration of 482.5 μM of NNK.

**Figure 4:**
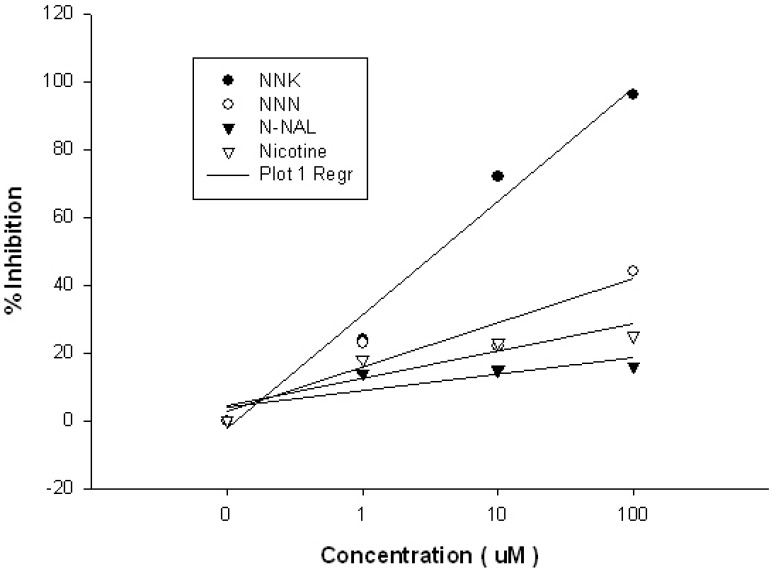
Relative affinity of mAb 4H2 for nicotine and other tobacco-specific nitrosamines (TSNAs). Competition Enzyme linked immunosorbent assays (ELISAs) were performed using NNK, NNN, *N*-NAL and nicotine as soluble competitors to determine the extent of inhibition when co-incubated with a 1:160 dilution of 4H2. Results showed that at 100 μM of free NNK, the greatest inhibition of 4H2 was seen (96%). No cross-reactivity of the antibody to nicotine or *N*-NAL was evident at any of the three varying concentrations. Forty-four percent inhibition of 4H2 occurred when incubated with 100 μM free NNN.

Splenocytes from the two mice with the strongest NNKB binding antisera were used for fusion with the Sp2/mIL-6 myeloma cell line. These cells constitutively express IL-6, which increases the number of hybridomas recovered from each fusion, thereby increasing the likelihood of recovering an NNKB-specific hybridoma [33]. This approach yielded over 600 hybridomas, nine of which were initially identified as NNK binders. Ultimately four hybridomas were characterized by competitive ELISA to assess their anti-NNK binding properties, with the antibody from the 4H2 hybridoma judged as superior based upon its affinity for the NNKB conjugates and 99% binding displacement when pre-incubated with excess free NNK prior to ELISA (Figure 3A,B). Subsequent competition studies showed that 4H2 was highly specific for NNK, with only moderate cross-reactivity with NNN, and virtually no binding affinity toward nicotine or N-NAL (Figure 4). 

**Figure 5:**
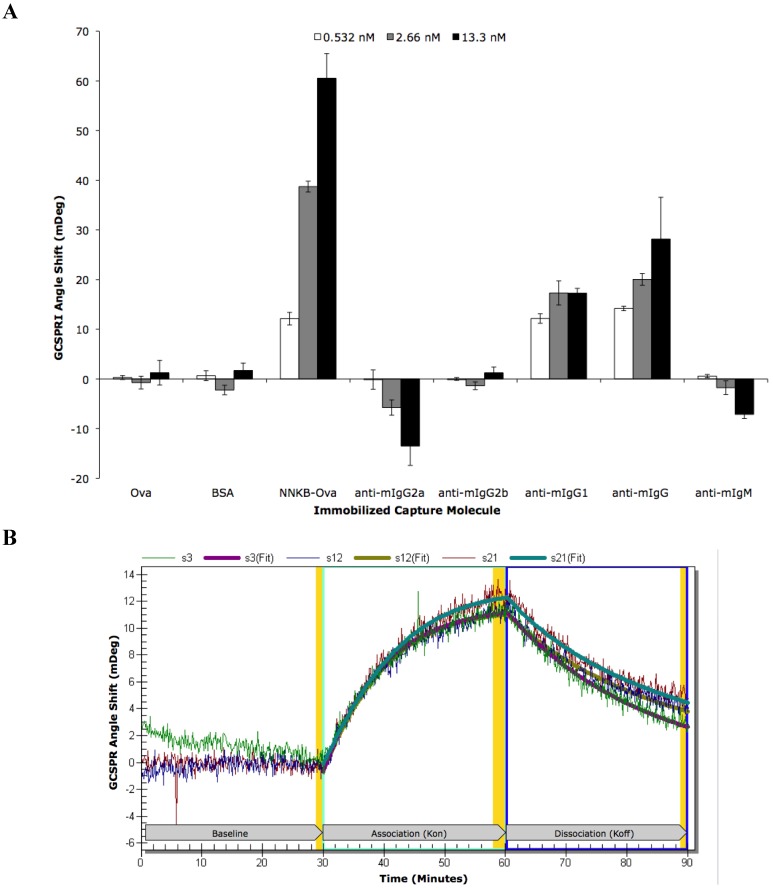
GCSPRI analysis of toxicant-specific antibody binding to a toxicant-protein adduct. NNKB-Ova was immobilized on five separate GCSPRI sensor chips along with appropriate negative controls and a series of murine Ig isotype-specific antibodies, all at a concentration of 500 μg/mL. (**A**) A different dilution of 7F mAb (0.532 nM, 2.66 nM, and 13.3 nM) was recirculated at 100 μL/min for 30 min over each of three identical sensor chips in three separate parallel experiments. GCSPRI angle shifts represent the mean of triplicate spots of capture molecules and error bars represent standard deviations; (**B**) Flexchip Kinetic Analysis Software was used to fit the real-time GCSPRI binding curves resulting from 7F mAb (0.54 nM) binding to triplicate spots of immobilized NNKB-Ova (500 μg/mL). Jagged lines represent raw (non-reference corrected) data for each of the three ROIs and smooth lines represent associated fitted curves. Affinity constants shown in Table 1 were determined using local kinetic analysis, fitting each of the triplicate curves individually, as well as global analysis of the triplicate spots together.

The 4H2 hybridoma was twice subcloned by limiting dilution and the resulting 7F hybridoma was used for all subsequent studies. GCSPRI analysis was utilized to further characterize specificity and affinity of the 7F mAb. These results demonstrated concentration-dependent binding of 7F mAb to immobilized NNKB-Ova and binding to anti-isotype specific antibodies suggested 7F to be of the murine IgG1 isotype (Figure 5A). Binding of 7F mAb to NNKB-Ova was inhibited by pre-incubation with free NNK (data not shown) and kinetic analysis revealed this to be a high affinity interaction (*K*_d_ = 2.93 nM) (Figure 5B and Table 1).

**Figure 6:**
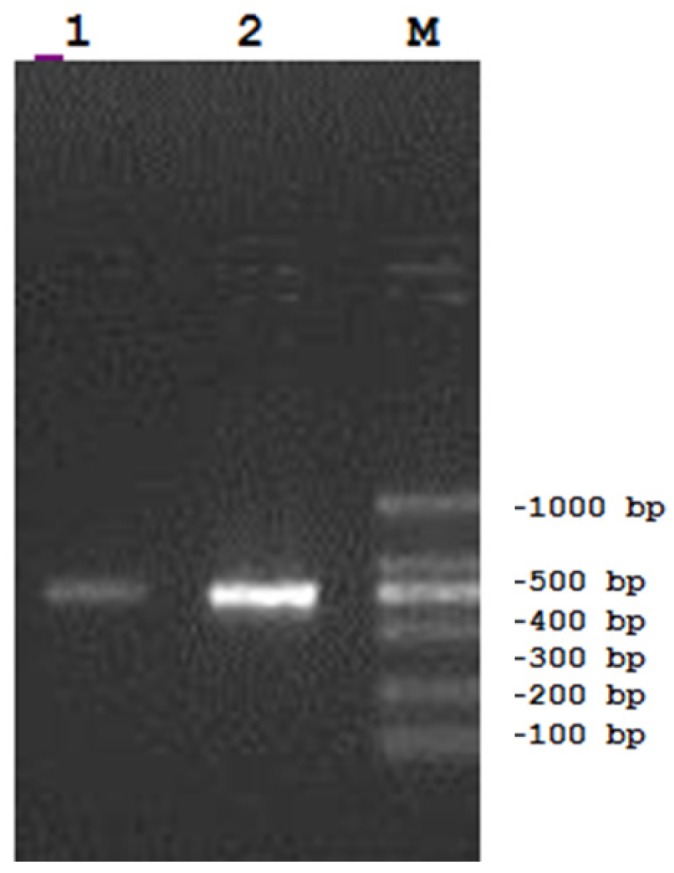
PCR amplification of the VH and VL Immunoglobluin chains of the 7F Mab. mRNA from the 7F hybridoma was converted to cDNA and then amplified via PCR. Using the appropriate primers, the variable heavy and light chains of the antibody were also amplified by PCR and then sequenced. Results showed two individual bands of the appropriate molecular weights. LANE 1: PCR amplification of the VL with the κ primers; LANE 2: PCR amplification of the VH with the Vh back primers and CH1 for primers; M: molecular weight standard.

**Figure 7:**
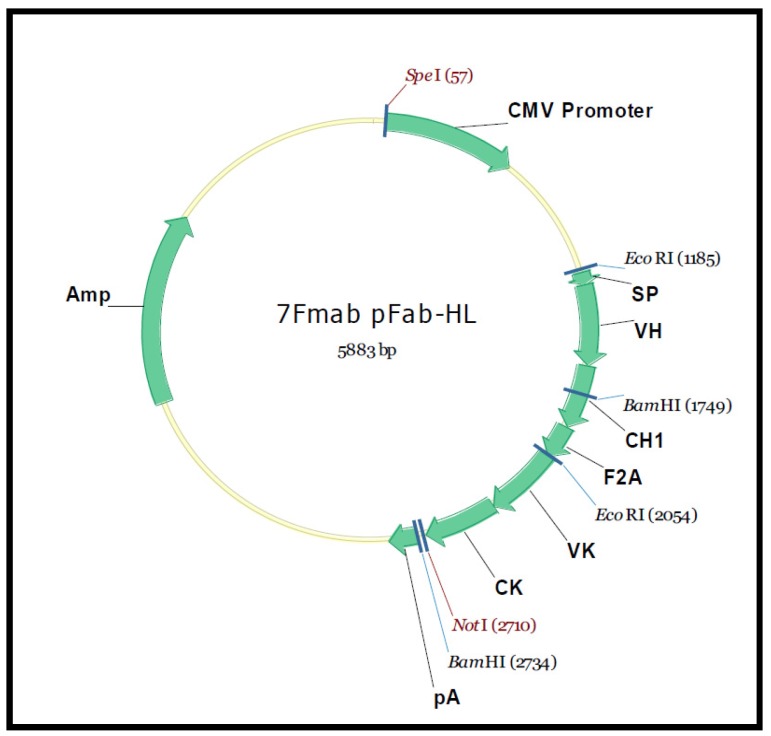
The schematic map of BSA-DP-pFab-H. The BSA-DP-pFab-H eukaryotic expression vector was used to express the recombinant anti-NNK fragment in HEK293 cells. The DNA of the 7F mAb Ig chains, which were linked by the 2A sequence were cloned into the vector via EcoRI/NotI.

**Figure 8:**
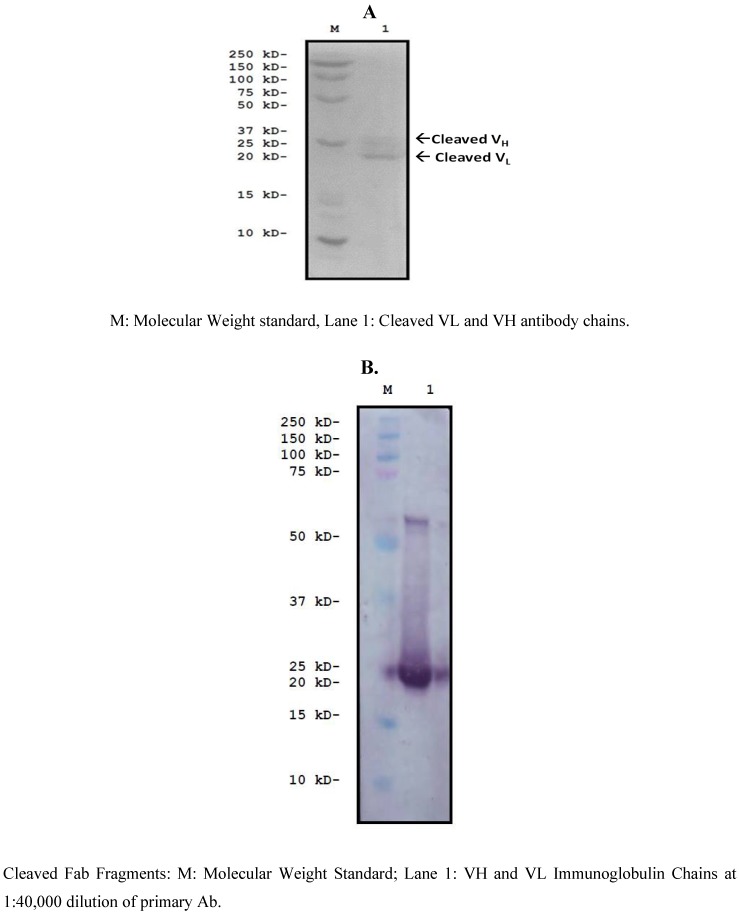
Characterization of Anti-NNK Binding Fragment. A non-denaturing SDS-PAGE (**A**) and Western Blot (**B**) revealed that expression of the F(ab) in HEK293 cells resulted in the successful cleavage of the fusion product into two individual immunoglobulin chains at the anticipated molecular weights.

**Figure 9:**
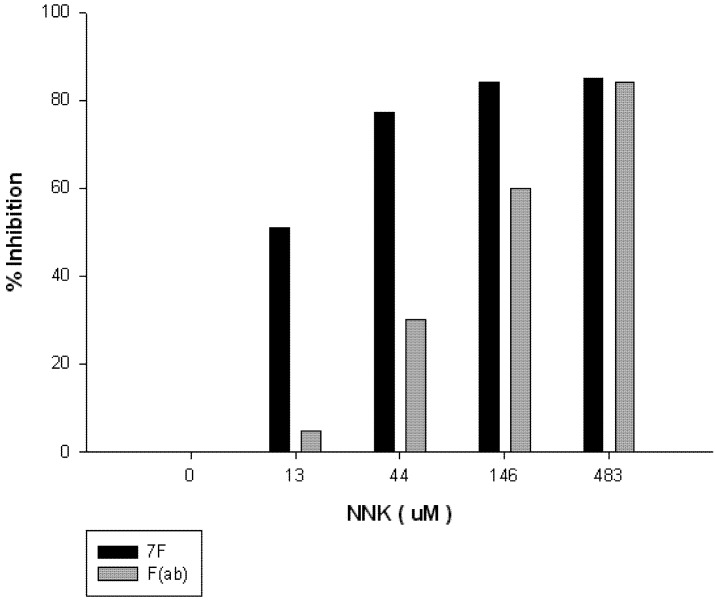
Competitive Inhibition of Anti-NNK Binding Fragment and 7F using free NNK. A 10 mg/mL stock solution of NNK was diluted to initial concentrations of 26 μM, 88 μM, 292 μM, and 966 μM. A 1:4 dilution of the F(ab) was added to each concentration of NNK to yield a final dilution of 1:8. Competition ELISA screening revealed that approximately 84% inhibition of the construct was seen at 483 μM of NNK. At the same concentration of NNK, 85% inhibition of the monoclonal antibody 7F was evident.

The mRNA from the 7F hybridoma VH and VL coding sequence was converted to cDNA, amplified, (Figure 6) sequenced and cloned into an expression vector with the FMDV 2A cleavage motif placed in-frame and between the two fragments (Figure 7). The resulting expression vector was used to transfect HEK293 cells to determine if a functional F(ab) was synthesized and secreted. Concentrated tissue culture supernatants were purified over an affinity column and the eluate analyzed by SDS-PAGE, which confirmed that the sequence was successfully translated, cleaved and secreted (Figure 8A). Western Blots confirmed the presence of the heavy and light chain fragments at the predicted molecular weights, however a stronger band was observed for the 24 kD fragment (Figure 8B). Competition studies using the anti-NNK binding fragment showed a concentration dependent inhibition of binding upon pre-incubation with free NNK, reaching 85% at the highest concentration tested (0.48 mM) (Figure 9).

**Table 1:** Regions of interest (ROI) data corresponding to Figure 5B.

ROI	ROI Content	Concentration	Kinetic Analysis	Kon	Koff	Ka (kon/koff)	Kd (koff/kon)
s3	NNKB-Ova	500 μg/mL	Local	1.94 × 10^6^	7.38 × 10^−4^	2.63 × 10^9^	3.80 × 10^−10^
s12	NNKB-Ova	500 μg/mL	Local	3.60 × 10^6^	8.18 × 10^−4^	4.40 × 10^9^	2.27 × 10^−10^
s21	NNKB-Ova	500 μg/mL	Local	3.76 × 10^6^	1.01 × 10^−3^	3.63 × 10^9^	2.75 × 10^−10^
All 3	NNKB-Ova	500 μg/mL	Global	2.87 × 10^6^	8.41 × 10^−4^	3.41 × 10^9^	2.93 × 10^−10^

## 3. Materials and Methods

### 3.1. Synthesis of 4-(4-Methylnitrosoamino-1-oxobutyryl)benzoic Acid (NNKB)

In order to produce a chemical moiety for coupling to carrier proteins, we synthesized a derivative bearing the identical methylnitrosoamino-1-oxobutyrate side chain, the target for antibody recognition on NNK, but substituted a benzoic acid ring structure in place of the pyridyl group. This approach served two functions; it allowed coupling to carrier proteins through the ring structure, leaving the desired *N*-nitroso group accessible to B-cell receptors; and it dimished the likelihood of inducing antibodies specific to nicotine or nicotinic B vitamins. 

Briefly, sodium hydride (60% in oil, 2.7 g, 67 mmol, 2 equiv.) was added to a dry flask and washed once with petroleum ether which was then removed. To this was added tetrahydrofuran (125 mL) followed by 1-methyl-2-pyrrolidinone (3.3 g, 33 mmol, 1 equiv.) dropwise. The resulting reaction mixture was stirred at room temperature for 1 h and dimethylterephthalate (9.7 g, 50 mmol, 1.5 equiv.) added as a solid in one portion. The flask was then immersed in a preheated oil bath (bath temperature 85 °C) and refluxed gently for 22 h. The reaction mixture was cooled to room temperature and acidified with HCl (2 N, 150 mL). The aqueous phase was extracted twice with dichloromethane and the combined organic layers dried over magnesium sulfate, filtered and evaporated. The residue was chromatographed on silica gel (gravity column, eluting with ethyl acetate:petroleum ether 1:1 then 4:1 with 1% methanol) to give a solid (4 g) which was recrystalized from ethyl acetate to give the product (2.6 g, 30%) as a mixture of keto-enol tautomers (the latter predominating) which did not give a sharp melting point. ^1^H NMR (CDCl_3_) δ 12.71 (s, 0.875H, enol form), 8.12–8.21 (m, 0.5H, enol form), 8.07 (d, *J* = 6.6 Hz, 1.75H, keto form), 7.74 (d, *J* = 6.5 Hz, 1.75H, keto form), 4.45 (m, 0.125H, enol form), 3.93 (s, 3H, both forms), 3.57–3.65 (m, 0.125H, enol form), 3.47 (t, *J* = 6.1 Hz, 1.75H, enol form), 3.36–3.46 (m, 0.125H, enol form), 2.99 (t, *J* = 6.1 Hz, 1.75Hz, enol form), 2.93 (s, 1.75H, enol form), 2.87 (s, 0.25H, keto form), 2.64–2.73 (m, 0.125H, keto form), 2.22–2.32 (m, 0.125H, keto form). The product of this series of steps is methyl 4-((1-methyl-2-oxopyrrolidine-3-yl)carbonyl)benzoate and is the structure shown on the far right in the line of Figure 1. A mixture of 4-((1-methyl)-2-oxopyrrolidine-3-yl)carbonyl)benzoate (2.3 g, 8.8 mmol) in HCl (5 N, 40 mL) was immersed in a preheated oil bath (bath temperature 105 °C) and stirred for 36 h. The bath temperature was then increased to 120 °C for a final 24 h. The solvent was evaporated to give a brown semi-solid, which was recrystallized from isopropanol/ethyl acetate to give the product as a solid (1.7 g, 75%). M.p. (with decomposition) 185–188 °C. ^1^H NMR (DMSO-d_6_) δ 8.18 (d, *J* = 8.2 Hz, 2H), 7.92 (d, *J* = 8.4 Hz, 2H), 4.36 (t, *J* = 7.9 Hz, 2H), 3.59 (t, *J* = 6.1 Hz, 2H), 3.64 (s, 3H), 2.38 (p, *J* = 7.8 Hz, 2H). The resulting 4-(4-methylamino-1-oxobutyryl)benzoic acid hydrochloride salt (middle structure of Figure 1) (1.4 g, 5.4 mmol, 1 equiv.) was dissolved in glacial acetic acid (16 mL), and stirred and cooled over ice. After five min, a solution of sodium nitrite (0.74 g, 11 mmol, 2 equiv.) in water (6 mL) was added drop-wise and the resultant homogeneous solution stirred over ice for 30 min and at room temperature overnight. The reaction mixture was diluted with water (100 mL) and extracted twice with ethyl acetate containing 5% methanol (2 × 100 mL) and CH_2_Cl_2_ (also 5% methanol, 100 mL). The combined organic layers were dried over sodium sulfate, filtered and evaporated to give a solid. This was recrystallized from dichloromethane/petroleum ether to give the product as white solid (*E*:*Z*, 4:1) (100 mg, 7%). M.p. 158–160 °C. ^1^H NMR (400 MHz, (CD_3_)_2_SO) *E* isomer: δ 10.12 (s, 1H), 8.11–8.02 (m, 4H), 4.21 (t, *J* = 7.0 Hz, 2H), 3.14 (t, *J* = 7.0 Hz, 2H), 3.02 (s, 3H), 2.07 (tt, *J* = 7.0, 7.0 Hz, 2H); *Z* isomer: δ 10.12 (s, 1H), 8.11–8.02 (m, 4H), 3.75 (s, 3H), 3.64 (t, *J* = 7.2 Hz, 2H), 3.01 (t, *J* = 8.7 Hz, 2H), 1.81 (tt, *J* = 7.1, 7.2 Hz, 2H); ^13^C NMR (100 MHz, (CD_3_)_2_SO) *E* isomer: δ 198.8, 166.6, 139.6, 134.5, 129.6, 128.0, 52.3, 35.0, 31.2, 21.8; *Z* isomer: δ 198.8, 166.6, 139.6, 134.5, 129.6, 128.0, 43.6, 38.6, 35.5, 19.6; IR ((CD_3_)_2_SO) 3421s, 1684s, 1542m, 1507m, 1457m, 1419m, 1338m, 1272m cm^-1^; MS (EI) *m/z* 250 (M^+^), 233, 220 (100), 149, 121, 73, 65; HRMS (FAB) calculated for C_12_H_15_N_2_O_4_ (M^+ ^+ H) *m/z* 251.1032. Found: 251.1026. The final product was 4-(4-methylnitrosoamino-1-oxobutyryl)benzoic acid (NNKB) which was used as the chemical mimic of NNK. The carboxylic acid and the *N*'-nitroso groups are the critical functional groups since the former will be used in the coupling reactions and the latter is what gives the compound its potential carcinogenicity. 

### 3.2. Chemical Coupling of NNKB to Carrier Proteins

Bovine serum albumin (Sigma-Aldrich Inc. St. Louis MO, USA) and ovalbumin (Sigma-Aldrich Inc., St. Louis, MO, USA) were selected as carrier proteins for the NNKB hapten. Briefly, 0.23 mM of 1-ethyl-3-(3-dimethylaminopropyl) carbodiimide-HCl (EDAC) (Sigma-Aldrich Inc., St. Louis, MO, USA) was dissolved in 1 mL of 0.05 M 2-(*N*-morpholino)ethanesulfonic acid (MES) buffer, pH 4.75 (Sigma-Aldrich Inc., St. Louis, MO, USA). This solution was added drop-wise with stirring to a solution of 0.23 mM of NNKB dissolved in 9 mL of dimethylformamide (DMF) (Sigma-Aldrich Inc., St Louis, MO, USA). The solution was allowed to stand in the dark for 60 min, at room temperature, with gentle stirring. 0.42 μmol of either bovine serum albumin or ovalbumin, were dissolved in 42 mL of 0.05 M cyclohexylaminoethane sulfonic acid buffer (CHES) (United States Biochemical Corporation, Cleveland, OH, USA), pH 9.5. Once the carrier was completely dissolved, the NNKB solution was added drop-wise, with gentle stirring and allowed to stand for 14 h in the dark, at 4 °C, then dialyzed against 1 liter of phosphate buffered saline (PBS), pH 7.4. The dialysate was checked every 8- to 12-h (λ = 343 nm) until the optical density <0.010. The retentate was stored in 200 μL aliquots at −20 °C until needed for protein quantification, substitution ratio calculation or antibody generation. A commercially available Griess reaction kit (Molecular Probes, Eugene, OR, USA) was used to assess the *N*-nitroso concentration present on each. Briefly, 66 μL of sulfanilic acid, 66 μL of *N*-(1-napthyl)ethylenediamine dihydrochloride, 50 μL of concentrated hydrochloric acid, 50 μL of NNKB conjugate plus 1766 μL of deionized water was used. A blank sample was prepared by substituting 50 μL of deionized water in place of the NNKB-conjugate. The nitrite standards were made up similarly using serial dilutions of 1 mM solution of nitrite solution. The resulting samples were heated to 90 °C in a water bath for 60 min to cleave the *N*'-nitroso group, producing nitrite ion. The samples were then cooled for 5 min to room temperature and incubated for 65 min before measurement at λ = 548 nanometers in triplicate, using a Shimadzu UV-1601 PC with UV probe software.

The carrier protein concentration was determined using the Bio-Rad protein assay (Bio-Rad, Richmond, CA, USA) following the manufacturer’s protocol using a BSA standard curve (two fold dilution ranging from 1400 to 200 μg/mL). After mixing and a 5-min incubation time (room temperature), the absorbance was measured at λ = 595 nanometers in triplicate using a Shimadzu UV-1601PC UV-visible spectrophotometer as described above. Finally, the molar substitution ratios of nitrosated NNKB product of each conjugate (as determined by Griess reaction) to carrier protein was calculated as a simple ratio. 

### 3.3. Antibody Generation in Mice

Female BALB/c mice weighing 19 to 21 g were obtained from Charles River (Charles River, Wilmington, MA, USA) and allowed to acclimate for two weeks, at which time they were ear-tagged and randomly assigned to groups. They were provided food and water *ad libitum* and maintained using 12-hour light and dark cycles in a controlled environment (20 °C and 63% relative humidity). All animal protocols were approved in advance by the University of Connecticut’s IACUC committee and conformed to NIH guidelines. The NNKB-carrier protein conjugates were used to vaccinate mice following formulation in adjuvant by adding the appropriate volume (for 100 μg of conjugate) to 500 μL of MPL + TDM +CWS adjuvant (Sigma-Aldrich Inc. St. Louis, MO, USA) as outlined in the manufacturer’s instructions. Each mouse received 50 μL subcutaneously, 50 μL intramuscularly and 100 μL intraperitoneally of the NNKB-carrier protein conjugates. Identical booster vaccinations were administered 28 days after the priming dose. All vaccinations and blood collections were performed on anesthetized animals using an inhalation chamber containing 2% to 2.5% isoflurane (Baxter, Deerfield, IL, USA). Blood was collected by retro-orbital puncture and drawn into heparin treated vacutainer tubes (Becton Dickinson Vacutainer Systems, Franklin Lakes, NJ, USA). Blood was collected one week before, and 2, 4 and 6 weeks after the priming vaccination. The blood was refrigerated at 4 °C for 30 min then centrifuged using a table-top centrifuge (Edison, NJ, USA). The plasma was then stored at −20 °C until needed.

### 3.4. Enzyme Linked Immunosorbent Assays (ELISAs)

Microtiter plates (Immulon I) were coated with NNK-Ova and Ova, each at a concentration of 10 μg/mL in 0.05 M sodium carbonate buffer (Sigma-Aldrich Inc. St. Louis, MO, USA), pH 9.6. The plates were wrapped in two layers of Parafilm™ and incubated at room temperature overnight, then stored at 4 °C thereafter. On the day of assay, the plates were warmed to room temperature, unwrapped and blotted dry onto paper towels. Each plate was washed 5 times with PTA buffer (0.05 M phosphate buffer saline (PBS) + tween 20 (Aldrich, Milwaukee, WI, USA) + 0.01% sodium azide (Fluka, Buchs Sg, Switzerland)), with 0.1% ovalbumin, pH 7.4 using a Biotek EL 403 microplate autowasher (Biotek Instruments, Winooski, VT, USA). The plates were blotted dry to remove any residual buffer from the wells, followed by the addition of samples. Each serum sample was serially two-fold diluted (ranging from 1:2000 to 1:256,000) and applied to the microtiter plate. The plates were wrapped in Parafilm™ and incubated for 3 h at 4 °C. The plates were then washed as described above, and 100 μL/well of secondary antibody diluted in PTA, pH 7.4 was applied at a 1:2000 dilution (goat anti-mouse alkaline phosphatase conjugated IgG, Fc specific; Sigma-Aldrich Inc., St. Louis, MO, USA). The plates were then wrapped as before and incubated overnight at 4 °C. The plates were then warmed, unwrapped, blotted dry, washed, and blotted prior to the addition of 100 μL of a 1 mg/mL solution of *p*-nitrophenyl phosphate (Sigma-Aldrich Inc., St. Louis, MO, USA and Kirkegaard, & Perry Laboratories, Gaithersburg, MD, USA) dissolved in 0.05 M sodium carbonate + 0.001 M magnesium chloride (Sigma-Aldrich Inc., St. Louis, MO, USA), pH 9.8. The plates were incubated at room temperature and absorbance readings were taken at 25, 50 and 100 min after the addition of substrate using a Bio-Tek EL311 microplate autoreader (Biotek Instruments, Winooski, VT, USA), λ = 405 nanometers. The data was captured using KCJr for Windows software (Biotek Instruments, Winooski, VT, USA).

### 3.5. ELISA of F(ab)

ELISA was performed to test the binding of the F(ab) to its target antigen (NNK). One 96-well plate was coated with 10 μg/mL NNKB-Ovalbumin and 10 μg/mL Ovalbumin. Each of the coating solutions were diluted in 0.05 M sodium carbonate buffer (Sigma-Aldrich Inc., St. Lous, MO, USA), pH 9.6. The plate was wrapped in two layers of Parafilm and incubated at room temperature (21 °C) overnight. The next morning, a 1:8 dilution of the protein was made in PTA/BSA 0.1% buffer. A 1:2500 dilution of 7F was also made up to test on the same plate in order to compare the binding efficacies of the complete IgG versus the anti-NNK binding fragment. After the solutions were mixed, the 96-well plate was washed five times with PTA/BSA 0.1% buffer using a Bio-Tek ELx405 Microplate Washer. The plate was then blotted dry on paper towels. Fifty microliters/well of each antibody was added to their corresponding locations within the plate. The plate was then placed at room temperature for overnight incubation. The following morning, the plate was washed three times in PTA/BSA 0.1% buffer and blotted dry as above. A 1:1,000 dilution of Polyvalent Anti-Mouse IgG, A, M antibody conjugated to Alkaline Phosphatase was made up for application to the wells containing the 7F antibody. A 1:10,000 dilution of anti-Mouse IgG (Fab-specific) antibody conjugated to Alkaline Phosphatase was made for application to the wells containing the F(ab). Fifty microliters of each solution was then added to the appropriate wells. After a two hour incubation at room temperature the plate was washed 3 times once again using PTA/BSA 0.1% buffer. Two 5 mg tablets of the phosphatase substrate PNPP (Sigma-Aldrich, Lot # 120N8201V, St. Louis, MO, USA) were diluted in 10 mL of 0.05 M sodium carbonate + 0.001 M magnesium chloride (Sigma-Aldrich Inc., St. Louis, MO, USA) pH 9.8 and 50 μL of this mixture was added to each of the wells. Results were then read at 25, 50, and 100 min using a Bio-Tek uQuant Universal Microplate Spectrophotometer set at a wavelength of 405 nm (Biotek Instruments, Winooski, VT, USA).

### 3.6. Competitive ELISA

Competitive ELISAs were performed using the week 6 sera from the two best responders in each group. Stock solutions of each competitor were made up at a concentration of 48.25 mM (10 mg/mL) in de-ionized water, and then diluted to final concentrations of 48.25, 4.825 and 0.4825 μM. 50 μL of each competitor were added to 50 μL of a 1:4000 dilution of Polyvalent Anti-Mouse IgG, A, M antibody conjugated to Alkaline Phosphatase and incubated for one hour prior to addition to the plate. The plate was then washed three times as above after a two hour incubation period with each solution was completed. The phosphatase substrate PNPP was added at a volume of 50 μL to each well. Results were read at 25, 50 and 100 min intervals using the Bio-Tek uQuant Universal Microplate Spectrophotometer set at a wavelength of 405 nm. Two structurally similar TSNAs were also tested to assess cross-reactivity, as was free nicotine. A *K*_i_ was calculated for those competitors that produced a concentration dependent decrease in specific antibody binding. 

### 3.7. Competitive ELISA of F(ab)

A competition ELISA using the F(ab) was performed with NNK as the soluble competitor. The monoclonal antibody 7F was used as a positive control. A stock solution of NNK was made at a concentration of 48.25 mM (10 mg/mL) in de-ionized water. This solution was then diluted to 966 μM, 292 μM, 88.0 μM and 26.0 μM. 125 μL of each dilution of NNK was added to separate test tubes, which contained 125uL of either a 1:4 dilution of F(ab) or a 1:1250 dilution of 7F. The samples were then mixed and incubated at room temperature for 30 min in order to allow for appropriate competition to occur. 50 μL of each test tube containing the samples were added to their corresponding wells. After a 2 h incubation period, plates were washed three times as above. A 1:10,000 dilution of anti-Mouse IgG (Fab-specific) antibody conjugated to Alkaline Phosphatase was then applied to the F(ab)-containing wells and a 1:1000 dilution of Anti-Mouse IgG, A, M antibody conjugated to Alkaline Phosphatase was applied to 7F-containing wells, each at a volume of 50 μL/well. Plates were then washed as above followed by PNPP substrate addition. Results were read at 25, 50 and 100 min intervals with the Bio-tek spectrophotometer set at a wavelength of 405 nm. 

### 3.8. Monoclonal Antibody Production

The hybridoma fusion was performed according to [34] with some minor revisions. Mouse splenocytes were combined with Sp2/mIL-6 (ATCC, CRL-2016™; Manassas, VA, USA) mouse myeloma cells (10^7^) in a sterile 50 cc conical tube containing 50 mL of Hybricare medium (ATCC, Manassas, VA, USA). Two fusions were performed using splenocytes from the two mice with the highest anti-NNK titers. Hybridoma supernatants serially diluted 1:16–1:1024 in PTA/BSA 0.1% were added to wells and incubated 2 h. ELISA screening was performed as described above, but using an anti-mouse polyvalent immunoglobulins (G,A,M) alkaline phosphatase (Sigma, St. Louis, MO, USA) specific secondary antibody since the antibody isotype and subclass is not known *a priori*. From more than 600 hybridomas, nine were initially identified as NNK binders, with four subsequently characterized by competitive ELISA to assess their anti-NNK binding properties. The 4H2 hybridoma was subsequently twice sub-cloned by limiting dilution (average cell density 0.33 per well) to assure monoclonality. Using the anti-mouse polyvalent Ig (G,A,M) alkaline phosphatase conjugated secondary antibody, ELISA screening was performed as above and clone 7F was selected based on high affinity binding to NNKB. Samples were placed in liquid nitrogen for later use.

### 3.9. GCSPRI Analysis of Monoclonal Antibody Characterization

GCSPRI sensor chips were prepared and GCSPRI assays were performed as previously described [35]. Gold-coated GCSPR sensor chips were obtained from Ciencia, Inc. (East Hartford, CT, USA). Sensor chips were cleaned using 70% ethanol in deionized, distilled water prior to use and allowed to air dry. Replicate spots of capture molecules (NNKB-Ova), anti-immunoglobulin isotype-specific antibodies, and irrelevant control molecules were deposited on the unmodified gold surface of GCSPRI sensor chips using a robotic microspotter and immobilized by passive adsorption. A SpotBot2 robotic spotter using an ArrayIt Stealth Micro Spotting Pin, size SMP7B (TeleChem International, Inc. Sunnyvale, CA, USA), was used to print microarrays configured as individual spots, printed in triplicate, of approximately 255 μm in diameter. Chips were incubated with the deposited capture molecules in a humid chamber for at least 30 min. and allowed to air dry prior to use. In some cases, spotted sensor chips were stored in a vacuum dessicator at 4 °C until use. 

Automated kinetic scans of individual regions of interest (ROIs) corresponding to immobilized capture molecules were performed using a Flexchip scanning GCSPRI instrument (GE Life Sciences, Piscataway, NJ, USA). PBS containing 0.5% Tween-20 (PBST) (Sigma-Aldrich Inc. St. Louis, MO, USA) was used as running buffer and as a diluent for blocking buffer and 7F mAb analyte. A syringe was used to deliver five cycles of blocking buffer (2% BSA in PBST), each cycle consisting of 5 mL blocking buffer with a 5 min pause between each. A peristaltic pump was used to pass running buffer (PBST) over the chip for 30 min to establish a pre-sample PBST baseline, followed by a 30 min association stage in which 2.5 mL of 7F mAb analyte (diluted in PBST) was re-circulated over the chip, and finally a 30 min PBST wash was performed (dissociation stage). All assays were performed at room temperature and specific assay conditions (such as analyte concentration and flow rates) are described in the figure legend. Flexchip Kinetic Analysis Software was used for both endpoint and kinetic data analysis. Both “local” kinetic analysis of individual ROIs and “global” kinetic analysis of replicate ROIs were performed with mass transport effects considered in calculation of affinity constants shown in Table 1. Non-specific binding to bare gold was subtracted from apparent binding to target ROIs to generate reference-corrected data. From this reference-corrected data, a specific end-point GCSPRI angle shift was quantifiable for each antibody spot; this shift is expressed as a change in millidegrees from pre-sample baseline to post-sample baseline. End-point GCSPRI angle shifts at replicate ROIs are indicated as mean ± standard deviation.

### 3.10. Cloning and Characterization of a F(ab)-Generating Single Chain Antibody

Four T-75 culture flasks containing 7F cells in Hybricare Media supplemented with MEM Sodium Pyruvate (100 mM), MEM Non-essential AA (10 mM), L-Glutamine (200 mM), 1× AB/AM and 20% heat inactivated FBS were sent to Creative Biolabs (Shirley, NY, USA) for amplification and sequencing. An intervening 2A motif derived from FMDV was cloned into the expression vector 7Fmab_pFab-HL via restriction enzymes EcoRI and NotI. The amplified VH and VL Immunoglobulin chains of the antibody flanked the peptide linker and a furin cleavage site was inserted just upstream of the 2A motif. The construct was transfected into HEK293 cells to allow for cleavage and subsequent expression of the recombinant binding fragment. Under denaturing conditions, an SDS-PAGE was performed according to [36] in order to verify cleavage and the appropriate molecular weight of the polyprotein. Next, a Western Blot was performed to test the protein’s immunoreactivity to a Fab-specific antibody. The primary antibody used was an Anti-Mouse IgG (Fab-specific) conjugated to alkaline phosphatase (Sigma-Aldrich, St. Louis, MO, USA) and diluted 1:10,000 in PTA/BSA 0.1% buffer (0.05 M phosphate buffer saline (PBS) + tween 20 (Aldrich, Milwaukee, WI, USA) + 0.01% sodium azide (Fluka, Buchs Sg, Switzerland)), with 0.1% ovalbumin added to the buffer pH 7.4. A second Western Blot was performed using a 1:40,000 dilution of the Anti-Mouse IgG (Fab-specific) antibody in order to better visualize the Immunoglobulin chains.

### 3.11. Statistical Analysis

A randomized complete block design was used with a statistical significance value set to *p* ≤ 0.05. The data was tested for Gaussian distribution using Kolmogorov-Smirnov test for normality using the PROC UNIVARITAE NORMAL command under SAS (SAS, Cary, NC, USA). The variances were tested for homogeneity using the Levene’s test for homogeneity of variance through the PROC GLM command under SAS. If the data were found to be normally distributed, and had homogeneous variances, the PROC ANOVA command was performed. When found to be significant a Tukey-Kramer mean separation post-hoc test was done. If the data failed either the Kolmogorov-Smirnov test or the Levene’s test, the Friedman non-parametric two-way analysis of variance was done. Additional statistical analysis was performed on data obtained from the F(ab) and 7F Competition ELISA. Using SAS 9.2 for Windows, the significance level α was set to 0.05% and a Mixed Procedure with a Diagonal Covariance Structure was performed. 

## 4. Conclusions

Immune-mediated sequestration of carcinogens or toxicants is an attractive strategy for mitigating the harmful biological effects of these compounds. Previous studies using monoclonal antibodies have mitigated the acute toxicity effects of drugs such as cocaine, heroin, methamphetamine, colchicine, oleander and other related substances [37,38,39,40]. Others have ameliorated the chronic toxicity associated with chemicals such as PCP, NNK and nicotine [17,41,42,43]. The current work builds upon these studies by creating a high affinity recombinant anti-NNK binding site derived from a mouse monoclonal antibody specific for NNK. 

The immunization of mice with an NNK mimic (NNKB) chemically conjugated to carrier proteins produced high-titered antibodies to NNK in serum, and subsequent splenic fusion gave rise to four hybridomas specific for NNK. Approximately 99% of the monoclonal antibodies from one candidate hybridoma (4H2) were competitively displaced from binding plates coated with NNKB conjugates, when co-incubated with increasing concentrations of NNK. The subclone 7F bound to NNKB-Ova conjugates with the highest affinity (*K*_d_ of 2.93 nM) based on GCSPR studies and was subsequently chosen for construction of a recombinant anti-NNK binding fragment. The VH and VL immunoglobulin chains were amplified from purified mRNA and cloned into an expression vector with a 2A peptide linker derived from FMDV inserted between the fragments. The incorporation of this self-cleaving peptide linker has been shown to significantly improve protein expression and is superior to bi-cistronic plasmids with two IRES sequences in terms of protein translation and a more compact coding sequence [25,44]. When the construct was expressed in HEK 293 cells, the resulting F(ab) fragment demonstrated high-affinity binding characteristics toward NNK, with about a one-log reduction in affinity from the parent monoclonal antibody. 

The decrement in affinity may have resulted from inefficient cleavage of the sequence downstream of 2A, which could affect proper folding of the immunoglobulin chains thereby interfering with binding to the target antigen. Access of the antigen (NNK) to one of the complementarity determining regions (CDRs) of the variable light chain may also have been blocked due to the close proximity of the signal peptide sequence to this region. Another reason may have been due to the fact that the *N*-terminus of the 2A sequence contained only five additional AAs from the 1D capsid protein of FMDV (GenBank Accession #KC496013). According to Donnelly *et al.* [45], extension of the *N*-terminus of the 2A sequence using 14AAs of 1D or longer increased cleavage efficiency and resulted in a 1:1 ratio of two protein products. The addition of only five AAs from 1D resulted in a 1.8 to 1 ratio of the upstream protein versus the downstream one. This finding was confirmed in studies performed by Donnelly [46] and Rottier [47] where incorporation of greater than five amino acids from the 1D capsid improved cleavage efficiency by at least 13%. This may have been the reason why in the current study the heavy chain from the 7F monoclonal antibody was secreted from the HEK293 cells at a higher level than the light chain (Figure 8b). 

The current work demonstrates the ability of a F(ab)-derived from an anti-NNK monoclonal antibody to successfully bind to the target carcinogen with high-affinity. A long-term goal of this proposal is to express this recombinant antibody binding site in transgenic tobacco plants in order to sequester NNK either in the plant during curing, or in the oral cavity of tobacco users following *N*-nitrosation of nicotine. An advantage of this approach includes the use of single chain antibodies as opposed to the parent hybridoma, which would allow for ease of cloning owing to its small size. Expressing recombinant antibody sites in tobacco is ideal because the maintenance of transgenic crops requires minimal resources and costs [48]. This is in contrast to the labor intensive measures needed to produce and purify monoclonal antibodies. In 2002, Kathuria *et al.* [49] was able to express a fully functional recombinant antibody against HCG, a heterodimeric glycoprotein, in tobacco leaves at levels of up to 40 mg pure protein per kg of fresh leaf weight. Subsequent studies have confirmed that transgenic plants can express functional recombinant antibodies and fragments [50,51,52]. The current work paves the way for novel tobacco harm reduction immunotherapeutics that may one day be marketed in order to provide a less hazardous product to long-term tobacco users or those who switch from a much more hazardous smoked product.

## References

[1] World Health Organization (1987). Overall evaluations of carcinogenicity: An updating of IARC Monographs volumes 1 to 42. IARC Monogr. Eval. Carcinog. Risks Hum. Suppl..

[2] Rodu B., Jansson C. (2004). Smokeless tobacco and oral cancer: A review of the risks and determinants. Crit. Rev. Oral Biol. Med..

[3] Chiang H.C., Huang Y.K., Chen P.F., Chang C.C., Wang C.J., Lin P., Lee H.L. (2012). 4-(Methylnitrosamino)-1-(3-pyridyl)-1-butanone is correlated with 8-hydroxy-2'-deoxyguanosine in humans after exposure to environmental tobacco smoke. Sci. Total Environ..

[4] Nilsson R. (2011). The molecular basis for induction of human cancers by tobacco specific nitrosamines. Regul. Toxicol. Pharmacol..

[5] Hecht S.S. (1999). Tobacco smoke carcinogens and lung cancer. J. Natl. Cancer Inst..

[6] Schuller H.M. (1989). Cell type specific, receptor-mediated modulation of growth kinetics in human lung cancer cell lines by nicotine and tobacco-related nitrosamines. Biochem. Pharmacol..

[7] Schuller H.M. (2007). Nitrosamines as nicotinic receptor ligands. Life Sci..

[8] Arredondo J., Chernyavsky A.I., Grando S.A. (2006). Nicotinic receptors mediate tumorigenic action of tobacco-derived nitrosamines on immortalized oral epithelial cells. Cancer Biol. Ther..

[9] Arredondo J., Chernyavsky A.I., Jolkovsky D.L., Pinkerton K.E., Grando S.A. (2006). Receptor-mediated tobacco toxicity: Cooperation of the Ras/Raf-1/MEK1/ERK and JAK-2/STAT-3 pathways downstream of alpha7 nicotinic receptor in oral keratinocytes. Faseb. J..

[10] Arredondo J., Chernyavsky A.I., Grando S.A. (2006). The nicotinic receptor antagonists abolish pathobiologic effects of tobacco-derived nitrosamines on BEP2D cells. J. Cancer Res. Clin. Oncol..

[11] Rodu B., Cole P. (1994). Tobacco-related mortality. Nature.

[12] Hatsukami D.K., Lemmonds C., Tomar S.L. (2004). Smokeless tobacco use: Harm reduction or induction approach?. Prev. Med..

[13] Rodu B., Phillips C.V. (2008). Switching to smokeless tobacco as a smoking cessation method: Evidence from the 2000 National Health Interview Survey. Harm Reduct. J..

[14] Silbart L.K., Rasmussen M.V., Oliver A.R. (1997). Immunoprophylactic intervention in chemical toxicity and carcinogenicity. Vet. Hum. Toxicol..

[15] Kosten T., Owens S.M. (2005). Immunotherapy for the treatment of drug abuse. Pharmacol. Ther..

[16] Rasmussen M.V., Silbart L.K. (1998). Peroral administration of specific antibody enhances carcinogen excretion. J. Immunother..

[17] Silbart L.K., Keren D.F. (1989). Reduction of intestinal carcinogen absorption by carcinogen-specific secretory immunity. Science.

[18] Baud F.J., Borron S.W., Bismuth C. (1995). Modifying toxicokinetics with antidotes. Toxicol. Lett..

[19] Moolten F., Capparell N., Boger E. (1978). Reduction of respiratory tract binding of benzo[a]pyrene in mice by immunization. J. Natl. Cancer Inst..

[20] Moolten F.L., Schreiber B., Rizzone A., Weiss A.J., Boger E. (1981). Protection of mice against 7,12-dimethylbenz(a)anthracene-induced skin tumors by immunization with a fluorinated analog of the carcinogen. Cancer Res..

[21] Adair C.D., Buckalew V.M., Graves S.W., Lam G.K., Johnson D.D., Saade G., Lewis D.F., Robinson C., Danoff T.M., Chauhan N. (2010). Digoxin immune fab treatment for severe preeclampsia. Am. J. Perinatol..

[22] Daniels J.R., Wessinger W.D., Hardwick W.C., Li M., Gunnell M.G., Hall C.J., Owens S.M., McMillan D.E. (2006). Effects of anti-phencyclidine and anti-(+)-methamphetamine monoclonal antibodies alone and in combination on the discrimination of phencyclidine and (+)-methamphetamine by pigeons. Psychopharmacology.

[23] Hicks M.J., De B.P., Rosenberg J.B., Davidson J.T., Moreno A.Y., Janda K.D., Wee S., Koob G.F., Hackett N.R., Kaminsky S.M. (2011). Cocaine analog coupled to disrupted adenovirus: A vaccine strategy to evoke high-titer immunity against addictive drugs. Mol. Ther..

[24] Deng W., Yang D., Zhao B., Ouyang Z., Song J., Fan N., Liu Z., Zhao Y., Wu Q., Nashun B. (2011). Use of the 2A peptide for generation of multi-transgenic pigs through a single round of nuclear transfer. PLoS One.

[25] Chan H.Y., V S., Xing X., Kraus P., Yap S.P., Ng P., Lim S.L., Lufkin T. (2011). Comparison of IRES and F2A-based locus-specific multicistronic expression in stable mouse lines. PLoS One.

[26] Verrier J.D., Madorsky I., Coggin W.E., Geesey M., Hochman M., Walling E., Daroszewski D., Eccles K.S., Ludlow R., Semple-Rowland S.L. (2011). Bicistronic lentiviruses containing a viral 2A cleavage sequence reliably co-express two proteins and restore vision to an animal model of LCA1. PLoS One.

[27] De Felipe P. (2004). Skipping the co-expression problem: The new 2A “CHYSEL” technology. Genet. Vaccines Ther..

[28] Reed C.D., Rast H., Hu W.G., Mah D., Nagata L., Masri S.A. (2007). Expression of furin-linked Fab fragments against anthrax toxin in a single mammalian expression vector. Protein Expr. Purif..

[29] Tan Y., Liang H., Chen A., Guo X. (2010). Coexpression of double or triple copies of the rabies virus glycoprotein gene using a “self-cleaving” 2A peptide-based replication-defective human adenovirus serotype 5 vector. Biologicals.

[30] Luke G., Escuin H., de Felipe P., Ryan M. (2010). 2A to the fore—Research, technology and applications. Biotechnol. Genet. Eng. Rev..

[31] Hagemeyer C.E., von Zur Muhlen C., von Elverfeldt D., Peter K. (2009). Single-chain antibodies as diagnostic tools and therapeutic agents. Thromb. Haemost..

[32] Pathak T., Thomas N.F., Akhtar M., Gani D. (1990). Synthesis of [4–2H2]-, (4*R*)[4–2H1]- and (4*S*)[4–2H1]-4-(methylnitrosamino)-1-(3'-pyridyl)-1-butanone, C-4 deuterated isotopomers of the procarcinogen NNK. Tetrahedron.

[33] Harris J.F., Hawley R.G., Hawley T.S., Crawford-Sharpe G.C. (1992). Increased frequency of both total and specific monoclonal antibody producing hybridomas using a fusion partner that constitutively expresses recombinant IL-6. J. Immunol. Methods.

[34] Goding J.W. (1980). Antibody production by hybridomas. J. Immunol. Methods.

[35] Marusov G., Sweatt A., Pietrosimone K., Benson D., Geary S.J., Silbart L.K., Challa S., Lagoy J., Lawrence D.A., Lynes M.A. (2012). A microarray biosensor for multiplexed detection of microbes using grating-coupled surface plasmon resonance imaging. Environ. Sci. Technol..

[36] LaemmLi U.K. (1970). Cleavage of structural proteins during the assembly of the head of bacteriophage T4. Nature.

[37] Rouan S.K., Otterness I.G., Cunningham A.C., Holden H.E., Rhodes C.T. (1990). Reversal of colchicine-induced mitotic arrest in Chinese hamster cells with a colchicine-specific monoclonal antibody. Am. J. Pathol..

[38] Safadi R., Levy I., Amitai Y., Caraco Y. (1995). Beneficial effect of digoxin-specific Fab antibody fragments in oleander intoxication. Arch. Intern. Med..

[39] Shen X.Y., Orson F.M., Kosten T.R. (2012). Vaccines against drug abuse. Clin. Pharmacol. Ther..

[40] Treweek J.B., Janda K.D. (2012). An antidote for acute cocaine toxicity. Mol. Pharm..

[41] De Buck S.S., Schellenberger M.T., Ensch C., Muller C.P. (2009). Effects of antibodies induced by a conjugate vaccine on 4-(methylnitrosamino)-1-(3-pyridyl)-1-butanone absorptive transport, metabolism, and proliferation of human lung cells. Int. J. Cancer.

[42] Pentel P.R., Dufek M.B., Roiko S.A., Lesage M.G., Keyler D.E. (2006). Differential effects of passive immunization with nicotine-specific antibodies on the acute and chronic distribution of nicotine to brain in rats. J. Pharmacol. Exp. Ther..

[43] Gentry W.B., Ruedi-Bettschen D., Owens S.M. (2010). Anti-(+)-methamphetamine monoclonal antibody antagonists designed to prevent the progression of human diseases of addiction. Clin. Pharmacol. Ther..

[44] Mizuguchi H., Xu Z., Ishii-Watabe A., Uchida E., Hayakawa T. (2000). IRES-dependent second gene expression is significantly lower than cap-dependent first gene expression in a bicistronic vector. Mol. Ther..

[45] Donnelly M.L., Hughes L.E., Luke G., Mendoza H., ten Dam E., Gani D., Ryan M.D. (2001). The “cleavage” activities of foot-and-mouth disease virus 2A site-directed mutants and naturally occurring “2A-like” sequences. J. Gen. Virol..

[46] Donnelly M.L., Gani D., Flint M., Monaghan S., Ryan M.D. (1997). The cleavage activities of aphthovirus and cardiovirus 2A proteins. J. Gen. Virol..

[47] Groot Bramel-Verheije M.H., Rottier P.J., Meulenberg J.J. (2000). Expression of a foreign epitope by porcine reproductive and respiratory syndrome virus. Virology.

[48] Schillberg S., Fischer R., Emans N. (2003). Molecular farming” of antibodies in plants. Naturwissenschaften.

[49] Kathuria S., Sriraman R., Nath R., Sack M., Pal R., Artsaenko O., Talwar G.P., Fischer R., Finnern R. (2002). Efficacy of plant-produced recombinant antibodies against HCG. Hum. Reprod..

[50] Barbi T., Drake P.M., Drever M., van Dolleweerd C.J., Porter A.R., Ma J.K. (2011). Generation of transgenic plants expressing plasma membrane-bound antibodies to the environmental pollutant microcystin-LR. Transgenic Res..

[51] Makvandi-Nejad S., McLean M.D., Hirama T., Almquist K.C., Mackenzie C.R., Hall J.C. (2005). Transgenic tobacco plants expressing a dimeric single-chain variable fragment (scfv) antibody against Salmonella enterica serotype Paratyphi B. Transgenic Res..

[52] Ramirez N., Rodriguez M., Ayala M., Cremata J., Perez M., Martinez A., Linares M., Hevia Y., Paez R., Valdes R. (2003). Expression and characterization of an anti-(hepatitis B surface antigen) glycosylated mouse antibody in transgenic tobacco (*Nicotiana tabacum*) plants and its use in the immunopurification of its target antigen. Biotechnol. Appl. Biochem..

